# The League of Mercy Garden Party at Marlborough House

**Published:** 1905-07-08

**Authors:** 


### HOSPITAL ADMINISTRATION.

### CONSTRUCTION AND ECONOMICS.

### THE LEAGUE OF MERCY GARDEN PARTY AT MARLBOROUGH HOUSE.

The garden party which, thanks to the gracious kindness of the Prince and Princess of Wales, has become an annual feature of the progress of the League of Mercy, was held at Marlborough House on July 1st. The majority of the guests were invited for four o'clock, but before this the Prince of Wales received the presidents and lady presidents of the League in the saloon of Marlborough House, when a report of the work of the League during the past year was read by Mr. J. Harrison, M.V.O., one of the honorary secretaries.

In acknowledging the receipt of a copy of the report for the year the Prince of Wales said:

It gives me great pleasure as Grand President again to express my personal thanks to you and to all those who have worked under you on behalf of the League of Mercy, whose organisation has been considerably extended throughout London and the home counties during the past year.

I am much gratified to learn from the report which has just been read that another most successful year has been completed, and that the large sum of £14,000 has been handed over to King Edward's Hospital Fund. This makes a total contribution of £46,000 during the six years that the League has been in existence.

This steady increase is indeed most satisfactory, but I am well aware that such results could not have been attained without much personal trouble and sacrifice of time on the part of the members of the League.

The Princess desires me to express her regret that she is unable to be present here to-day, and in her name, as well as my own, I desire to once more thank you for the splendid work you have done on behalf of the League.

After the meeting, the party adjourned to the garden, where there were assembled about a thousand vice-presidents and members of the League. As his Royal Highness appeared on the steps leading from the house, the band of the Royal Marines, which was in attendance, played the National Anthem. The health of the Princess of Wales prevented her being present, and the guests did not expect the gathering to be graced by the presence of royal ladies. It was, therefore, a pleasant surprise when it was found that the Prince was accompanied not only by Princes Edward and Albert of Wales, but also by the Princesses Victoria and Louise of Schleswig-Holstein, who were attended by Mrs. Dick Cunyngham, while, soon after the ceremony of conferring the order of the League of Mercy had commenced, the royal party was re-inforced by Prince and Princess Alexander of Teck, whose interest in the work of the League may have been heightened by the fact that the Prince has recently accepted the post of President for the Kingston-Surbiton (with Wandsworth-Putney) Division.

London and the home counties are now nearly covered with a network of districts, presided over by presidents and vice-presidents, both ladies and gentlemen, numbering nearly 1,400, who organise the work of the 20,000 members of the League. The associates of the League (that is, subscribers of a shilling a year and upwards) now number some 300,000 men, women, and children. The fact that the contribution of a shilling makes the poorest as closely connected with the League as a donation of a larger sum, has brought into it many thousands of the humbler classes of the metropolis, who otherwise would never have dreamed of being thus drawn into connection with the King (the Patron) and the Prince and Princess of Wales (the Grand Presidents) of the League. The recent appointments to the League include the following presidents and vice-presidents:—Kingston and Surbiton District, H.S.H. Prince Alexander of Teck, K.C.V.O., D.S.O.; Chertsey, Sir Edward D. Stern and Lady Hooker; Croydon, Brigade-Surgeon Lieut.-Col. Henry G. Thompson, Esq., M.D., J.P., and Lady Edridge; North Camberwell and West Newington, Charles H. Hoare, Esq.; Clapham, Mrs. Thomas F. Rider; Dorking, Mrs. Leopold Salomons; Enfield, Rev. H. Howel Brown, M.A.; Horsham, C. J. Lucas, Esq., and Mrs. Maudslay; Saffron Walden, Mrs. Carl Meyer; Streatham, the Rev. H. Heneage Jebb, M.A.; Willesden, Dr. and Mrs. Carson Smyth; Woolwich, Lieut.-Col. W. Narborough, V.D.; Worthing, Mrs. Leicester Keppel.

#### The Order of Mercy.

During the last year the following ladies and gentlemen were awarded the Order of Mercy, with the sanction of his Majesty the King, and they were duly invested with it on the 1st inst. by H.R.H. the Prince of Wales.

Dora, Countess of Chesterfield. (Lady President, South Kensington District.)
Miss Harington. (Lady President, Bethnal Green District.)
Mrs. Hodson. (Lady President, West Islington District.)
Frederick Green, Esq. (President, Romford District.)
Mrs. Edmunds. (Lady Vice-President, Lambeth (Norwood) District.)
Mrs. B. G. Elliott. (Lady Vice-President, North St. Pancras District.)
Miss Grace L. M. Elliott. (Lady Vice-President, Woolwich District.)
Mrs. Amyas Longstaffe. (Lady Vice-President, South Paddington District.)
Mrs. Rodwell. (Lady Vice-President, Fulham District.)
Mrs. D. H. Scott. (Lady Vice-President, Richmond District.)
Daniel Hines, Esq. (Vice-President, Deptford-Brockley District.)
G. H. Judd, Esq. (Vice-President, Brentford District.)
A. W. Mellersh, Esq. (Vice-President, Guildford District.)
J. Seed, Esq. (Vice-President, North St. Pancras District.)
Councillor W. Sharp. (Vice-President, Eastbourne District.)
Philip F. Walker, Esq. (Vice-President, Fulham District.)
Miss Rosetta Abrahams. (Member, Whitechapel District.)
Mrs. G. Angus. (Member, Tonbridge District.)
Mrs. Balck. (Member, North St. Pancras District.)
Miss Bates. (Member, Maldon District.)
Miss Ida Belfield. (Member, Fulham District.)
Miss Mary Buckle. (Member, Lambeth (Norwood) District.)
Miss Chancellor. (Lady Vice-President, Richmond District.)
Mrs. Douglas Clark. (Member, Sevenoaks District.)
Miss Janet Campbell-Colquhoun. (Member, Sevenoaks District.)
Mrs. W. Cowen. (Member, Lewisham District.)
Miss Cullen. (Member, Croydon District.)
Mrs. Davenport. (Member, South Paddington District.)
Miss Margaret Dove. (Member, St. George, Hanover Square District.)
Mrs. E. Hay Forbes. (Member, Tonbridge District.)
Miss Betton-Foster. (Member, South Kensington District.)
Mrs. Gamble. (Member, South Paddington District.)
Miss Hickman. (Member, East Marylebone District.)
Miss Hilda Jones. (Member, South Kensington District.)
Miss Vera Johnston. (Member, Sevenoaks District.)
Mrs. Laurie. (Member, Sevenoaks District.)
Mrs. Le Marchant. (Member, Fulham District.)
Miss MacNiell. (Member, South Kensington District.)

Mrs. Mather. (Member, Epping District.)
Miss B. E. Narborough. (Member, Woolwich District.)
Miss Page. (Member, Chelsea District.)
Mrs. Parker. (Member, Battersea District.)
Miss Piggot. (Member, Maldon District.)
Miss Stella Pitt. (Member, Medway District.)
Miss Roberts. (Member, Richmond District.)
Mrs. G. W. Simpson. (Member, Tonbridge District.)
Mrs. A. M. Speer. (Member, Epsom-Esher District.)
Miss G. Todd. (Member, Richmond District.)

Besides these there were present many members of the League, including the Duchess of Somerset, the Duke of Argyll, Lord and Lady Wolverton, Lady Cadogan, Lady Llangattock, Lady Crossley, Lady Faudel Phillips, Sir Henry and Lady Burdett, and Sir William and Lady Collins. After the recipients of the order had been duly invested by the Prince of Wales, who shook hands with each, the royal party moved about among their guests, speaking to many of them and graciously acknowledging the salutations of all. Refreshments were served on the lawn, the royal party and a limited number of special guests being served in the royal tent, which was set amid a mass of white and pink hydrangeas, pelargoniums, and maiden-hair fern, while the rest of the company were provided for in a spacious marquee, the posts of which were wreathed with green leaves, while the table decorations consisted of alternate bouquets of roses and tall silver vases of white lilies. The band of the Royal Marines meanwhile discoursed enjoyable operatic selections until, at six o'clock, the strains of "God Save the King" intimated that the function was over and the members of the League of Mercy left the royal precincts to begin another year's work on behalf of their widespread and beneficent organisation.

